# Abnormal Population Responses in the Somatosensory Cortex of Alzheimer’s Disease Model Mice

**DOI:** 10.1038/srep24560

**Published:** 2016-04-15

**Authors:** Yossi Maatuf, Edward A. Stern, Hamutal Slovin

**Affiliations:** 1 The Mina and Everard Goodman Faculty of Life Sciences, Bar-Ilan University, Ramat-Gan 5290002 Israel; 2 The Gonda Multidisciplinary Brain Research Center, Bar-Ilan University, Ramat Gan, 5290002 Israel; 3 MassGeneral Institute of Neurodegenerative Disease, Department of Neurology, Massachusetts General Hospital, Charlestown, Massachusetts 02129, USA.

## Abstract

Alzheimer’s disease (AD) is the most common form of dementia. One of the neuropathological hallmarks of AD is the accumulation of amyloid-β plaques. Overexpression of human amyloid precursor protein in transgenic mice induces hippocampal and neocortical amyloid-β accumulation and plaque deposition that increases with age. The impact of these effects on neuronal population responses and network activity in sensory cortex is not well understood. We used Voltage Sensitive Dye Imaging, to investigate at high spatial and temporal resolution, the sensory evoked population responses in the barrel cortex of aged transgenic (Tg) mice and of age-matched non-transgenic littermate controls (Ctrl) mice. We found that a whisker deflection evoked abnormal sensory responses in the barrel cortex of Tg mice. The response amplitude and the spatial spread of the cortical responses were significantly larger in Tg than in Ctrl mice. At the network level, spontaneous activity was less synchronized over cortical space than in Ctrl mice, however synchronization during evoked responses induced by whisker deflection did not differ between the two groups. Thus, the presence of elevated Aβ and plaques may alter population responses and disrupts neural synchronization in large-scale networks, leading to abnormalities in sensory processing.

Alzheimer’s disease (AD), the most common form of dementia, is a degenerative disorder of the brain that is characterized by a progressive decline of cortical functions. Accumulation of extracellular amyloid plaques, composed of aggregated β-amyloid (Aβ) peptide, is one of the major neuropathological hallmarks of AD. Recent progress supports the amyloid hypothesis as a central trigger to induce a cascade of events leading to the clinical pathology seen in AD^1^,^2^. Various neural damage has been reported at the vicinity of Aβ plaques including synapse loss^3^,^4^, dystrophies of dendrites and axons^5^,^6^,^7^, and decrease in dendritic arborizations^8^. The curvature of neuronal processes was shown to increase near amyloid plaques^9^, and this was correlated with decreased reliability of synaptic response^10^. Although the effects of Aβ on function of synapses have been investigated extensively^11^,^12^, only few studies have focused on the effects of Aβ on neuronal circuits or complex spatiotemporal dynamics of neuronal networks at the mesoscopic scale with a millisecond time resolution^13^,^14^.

Abnormal neuronal hyperexcitability and epileptic seizure activity have been described in the frontal cortex and the hippocampal networks of transgenic (Tg) mice that showed high levels of Aβ plaques^15^,^16^,^17^. Recently, it has been reported that Aβ plaques induce hyperactive spontaneous activity in the frontal cortex of Tg mice^18^ and clusters of hyperactive neurons have been detected near the Aβ plaques^19^. Progressive accumulation of Aβ plaques is correlated with significantly reduced orientation and direction tuning performance of visual cortical neurons and a major source for this loss of tuning are the hyperactive neurons^20^. Moreover, a functional link between morphological degeneration of neurons and hyperexcitability was identified in Tg mice^21^. Finally, it has been suggested that abnormal synchronization levels between large cortical networks could be one of the causes of the cognitive impairments during the course of AD^22^. These results suggest that neural network dysfunction may play an important role in AD^13^,^23^,^24^. However, how this dysfunction of the cortical network affects the cortical processing of sensory inputs remains to be explored.

To investigate the effects of Aβ accumulation and deposition in the cortex, we performed voltage sensitive dye imaging (VSDI) in the barrel cortex of a well characterized mouse model of AD. VSDI enables simultaneous high spatial and temporal resolution (tens of micrometers per pixel; frame duration of ms) and high sensitivity to subthreshold synaptic potentials of populations of cortical neurons^25^,^26^,^27^,^28^. We hypothesized that in AD transgenic mice, aggregation of Aβ may induce disruption of functional columnar processing^23^,^24^,^29^. In this study, we imaged *in vivo* sensory evoked activity of large-scale networks in the barrel cortex of aged Tg mice and of non-transgenic control (Crtl) mice. We found that in Tg mice, whisker deflection evoked abnormal sensory responses in the barrel cortex and the spatial extent of the cortical response was significantly larger compared to Crtl mice. Finally, Tg mice showed changes in cortical synchronization, with a significant reduction in neuronal synchrony during spontaneous activity.

## Results

To investigate the impact of Aβ deposition on cortical processing we imaged population responses evoked by whisker stimulation in the barrel cortex of transgenic (Tg) mouse model of AD and their age-matched non-transgenic littermates controls (Ctrl). We used VSDI which enables *in-vivo* recording of population activity, emphasizing sub-threshold synaptic potentials at high spatial and temporal resolution across cortical areas. The dye signal represents the sum of membrane potential changes of all neuronal elements, including dendrites somta and axons, within each pixel in the imaged area. Thus, the measured signals reflect the population responses rather than responses of single neurons^27^,^28^,^30^,^31^.

**Figure 1:**
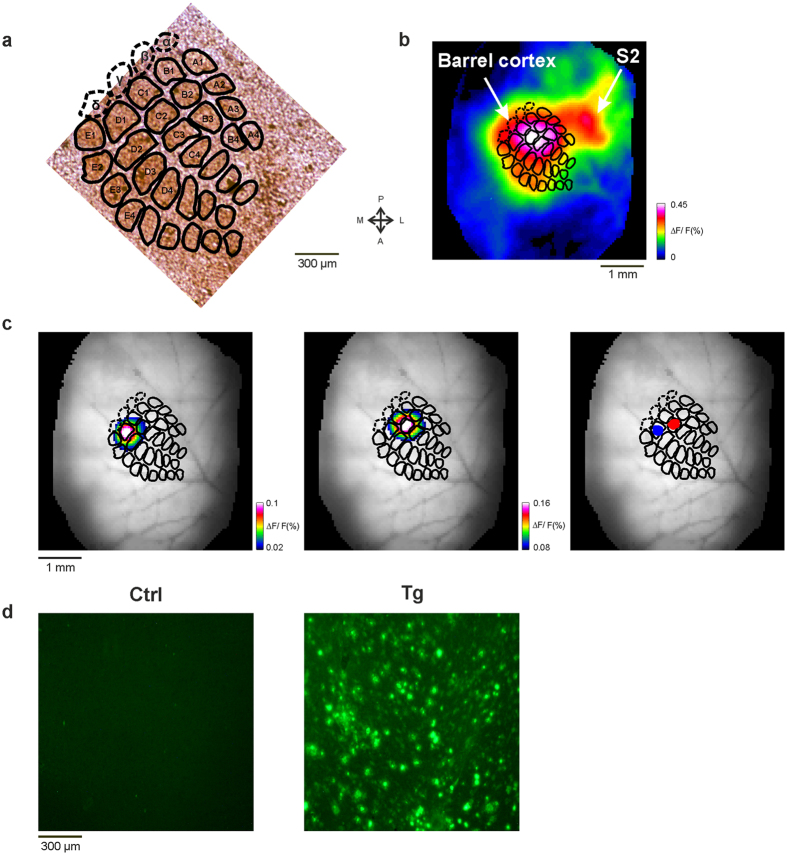
Mapping barrel fields in the barrel cortex. (**a**) Example of barrel columns, stained by cytochrome oxidase histochemistry, in layer IV of the somatosensory cortex of a Crtl mouse. Barrel fields are labeled for identification (dashed lines represent the location of alpha, beta, gamma and delta barrels, extrapolated from other experiment). Scale bar, 300 μm. (**b**) VSD response map of a Ctrl mouse brain, imaged at 40 ms after stimulus onset, showing the spread of activation in the barrel cortex after C2 whisker deflection. Blood vessels are shown in gray. (n = 20 trials). (**c**) Example session: VSD response maps (∆F/F) evoked by D1 (left map) and C2 (middle map) whisker deflection, superimposed on the barrel columns map from A. Maps were measured at 20 ms after stimulation onset and averaged across trials (n = 20 trials for each condition). The schematic representation of the barrel columns from A, shown as black contour lines, are scaled and superimposed over the blood vessels pattern. Right map: The colored areas indicate the selected ROIs of whiskers C2 and D1 (red and blue, respectively). (**d**) Example of horizontal sections of layer II/III in the primary somatosensory area of Tg (right) and Ctrl (left) mice. Tissues were stained with Thioflavine-S (see Methods). The plaques are seen as bright green spots.

As a first step, we verified that the VSD signals evoked by brief whisker deflection were localized to the barrel cortex. Cytochrome oxidase (CO) histochemistry in layer IV of the somatosensory cortex revealed the morphological organization of the barrel cortex (Fig. 1a). Previous imaging and electrophysiological studies have shown that in wild-type mice, the deflection of a single whisker evoked a short latency cortical response (10–20 ms after stimulation onset) that is localized to the barrel column of the contralateral stimulated whisker^31^,^32^,^33^,^34^,^35^,^36^. We applied a similar approach and used the VSD maps obtained in Ctrl mouse at 20 ms after stimulus onset (Fig. 1c) to demonstrate the VSD response in a single barrel-related column. The color coded VSD maps reflects the fluorescence intensities, generated by changes in membrane potential of neuronal populations. The response patterns evoked by whiskers D1 and C2 were located spatially on different sites and are shown in the left and middle maps of Fig. 1c. By superposing the barrel field outlines from Fig. 1a, we confirmed that the early high activity was restricted to the corresponding barrel columns in the cortex. Based on these maps, the region of interest (ROI) was defined as pixels exceeding an amplitude threshold at the activated area (C2 and D1 ROIs are shown as blue and red areas in Fig. 1c, right map; see also Methods). Thus, as expected, deflection of different whiskers triggers spatially distinct responses, which are well aligned with the anatomical barrel map obtained by CO staining. Fig. 1b shows that in the following time (>30 ms after stimulation onset) and in accordance with previous studies, the VSD response increased in amplitude and spread horizontally into surrounding whisker columns covering much larger area than the size of a single barrel^31^,^32^,^33^,^34^,^35^. The VSD map of Crtl mouse brain, obtained 40 ms after whisker deflection demonstrates the spread of activation to adjacent barrel columns in the barrel cortex (Fig. 1b). Additionally, whisker stimulation triggers responses in functionally related areas such as secondary somatosensory cortex (S2).

**Figure 2:**
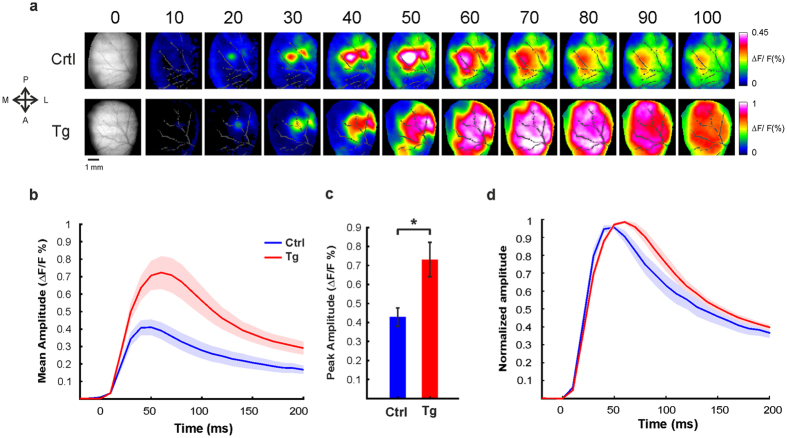
Whisker deflection evoked much higher response amplitude in the barrel cortex of Tg mice. (**a**) Population response maps, evoked by single brief whisker deflection, in the barrel cortex of Ctrl (top; example session, n = 20 trials) and Tg (bottom; example session, n = 20 trials) mice. The numbers above the maps represents the time in ms after whisker stimulation onset. Note the colorbar on the right and ∆F/F range that is much larger for the Tg mouse. Blood vessels are marked in gray. The VSD maps were low-pass filtered with a 2D Gaussian filter (σ = 1.5 pixels) for visualization purposes only. (**b**) Time course of the VSD response, grand average analysis. Time course of the VSD response in C2 ROI of Crtl and Tg mice (ROIs are demonstrated in Fig. 1c, right map). The responses were averaged across mice (n = 6 Tg mice; n = 8 Ctrl mice). Whisker stimulation is at t = 0. Shaded areas represent ± 1SEM (**c**) Peak response amplitude, grand average analysis. Peak response amplitude evoked by single brief whisker deflection, averaged across all animals (Tg; n = 6 mice, Crtl; n = 8 mice). *p < 0.05, Wilcoxon rank sum. (**d**) Normalized time course of responses, grand average analysis. The response was normalized to peak response amplitude of each mice and averaged across mice (n = 6 Tg mice; n = 8 Ctrl mice). Shaded areas represent ± 1SEM.

Next we wanted to compare the population response in the barrel cortex of Ctrl and Tg mice and first verified the accumulation of Aβ plaques in the barrel cortex of Tg mice (Fig. 1d, right; see Methods). To compare the cortical spatio-temporal patterns evoked by whisker stimulation in Tg mice to those in Ctrl mice, we measured a sequence of population-response maps evoked by brief deflection of C2 whisker. Example sessions are shown in Fig. 2a. The maps show that the early response was spatially localized and looked similar in the Ctrl and Tg animals (Fig. 2a maps at t = 20 ms; see also additional analysis below). However, at later times, when the population response rapidly spread over the barrel cortex and also to adjacent cortical areas, the Tg mouse showed distinctly different activity patterns from those observed in the Ctrl mouse. To quantify these results, we computed the grand average time course (across animals) of the VSD signal in the C2 ROI at the barrel cortex of Tg and Ctrl mice (Fig. 2b; see Methods). The population response showed similar latency in both groups, however, at later time the response in the Tg mice showed a much higher amplitude. The peak response in the Tg mice evoked by a single brief deflection of one whisker was higher by ~70% than the peak response in Crtl mice. Figure 2c depicts the grand analysis of the peak response for the two groups and shows that the peak response was significantly higher in Tg mice (mean ± sem; 4.3 × 10^−3^ ± 4.8 × 10^−4^ and 7.3 × 10^−3^ ± 9.1 × 10^−4^ for Ctrl (n = 8) and Tg mice (n = 6) respectively; *p < 0.05, Wilcoxon rank sum). To study the dynamics of the evoked response and compare it between Tg and Ctrl mice, we normalized the VSD time course to its peak response. This analysis enabled us to compare the response differences in the temporal domain, regardless of the peak amplitude (Fig. 2d). In Crtl mice, the activation arrived to peak response ~50 ms after stimulation onset, and then monotonically declined back to baseline. These results are consistent with previous studies^35^,^36^,^37^,^38^. Figure 2d shows that the population peak response in Tg mice had a delay of ~10 ms compared with the Ctrl mice (Fig. 2d), however this delay was not statistically significant (mean ± sem; 48.75 ± 7.96 and 60 ± 6.32 ms for Ctrl (n = 8) and Tg (n = 6) respectively; p = 0.0726, Wilcoxon rank sum). In addition, in the rising phase, the population response in Ctrl mice show faster dynamics than the Tg mice group (Fig. 2d). The max derivative of the normalized time course in the Ctrl group was higher than in the Tg group (0.43 ± 0.02 and 0.34 ± 0.01 for Ctrl (n = 8) and Tg (n = 6) respectively; p < 0.05, Wilcoxon rank sum). However, the min derivative value in the descending phase did not significantly differ between the groups (mean ± sem; −0.102 ±0.009 and −0.094 ± 0.006 for Ctrl (n = 8) and Tg (n = 6) respectively; p = 0.75, Wilcoxon rank sum).

**Figure 3:**
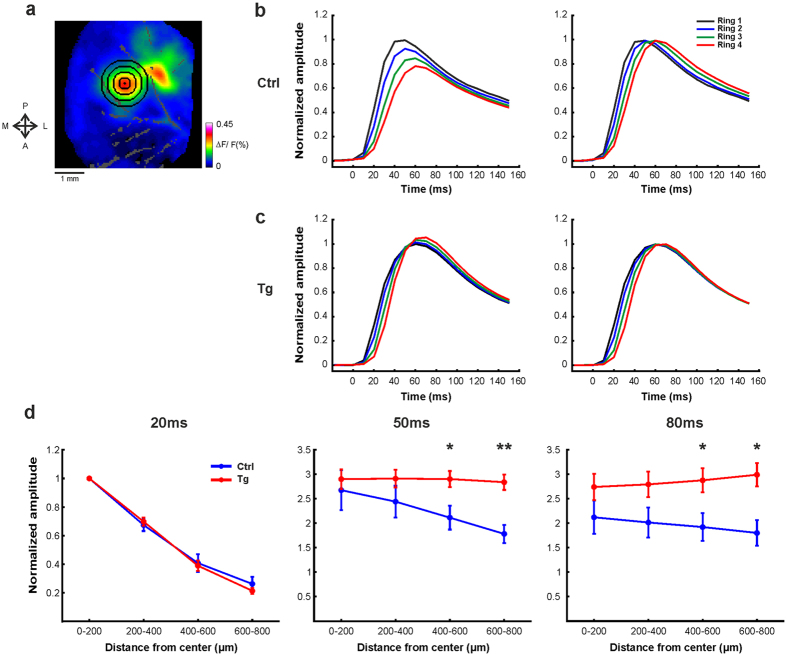
The spatial extent of the evoked response was significantly larger in Tg mice. (**a**) Schematic illustration of ring ROIs. The rings are four pixels wide (200μm) and are centered on the peak activation in space. The distance thus varies from zero (center of the rings) to 0.8 mm away from the center. The ring ROIs are numbered in ascending order according to their distance from the center of the barrel field (e.g. inner ring no. 1, most outer ring, no. 4). (**b–c**) Left: normalized time course response for each ring averaged over all pixels in the corresponding ROI. The response in each ring was normalized to the peak response of the inner ring. Whisker stimulation is at t = 0. Right: the time course in each ring was normalized to peak response. This highlights the differences in the latency to peak response between the rings. (**d**) The normalized amplitude, at 20, 40 and 80 ms after stimulation onset, as a function of the distance from the barrel center. Error bars represent ± 1SEM across mice (n = 6 Tg mice; n = 8 Ctrl mice). *p < 0.05 , Wilcoxon rank sum.

Next we wanted to investigate the spatial spread of the neural responses in both mice groups. For this purpose, we used a ring analysis approach (see schematic illustration Fig. 3a and Methods), where the cortical site with the highest response amplitude at the initial phase (20 ms after stimulation onset) was determined as the center point of the activated area (marked by the black dot in Fig. 3a). We then divided the area around the center into 4 non-overlapping ring ROIs, the width of each ring was 200 μm size and they extended up to 800 μm radius from the center (Fig. 3a). We computed the time course of the population response for each ROI separately by averaging the VSD signal over all pixels in each ring ROI. The responses were normalized to peak responses measured in the first, most central ring. Figure 3b shows the grand average analysis, for Ctrl (n = 8) and Tg mice (n = 6). In Crtl mice, the population response amplitude gradually decreased and response latency increased as the distance from the center increased (Fig. 3b, left). Moreover, the time to peak response was slightly shorter at the inner rings’ ROIs, as compared with the outer rings’ ROIs. To further investigate this, the time course response at each ring was normalized to its peak response value, thus emphasizing the difference between the peak response latency of each ring (Fig. 3b, right; time-to-peak for inner to outer rings: mean ± sem; 48.8 ± 3.9, 53.75 ± 4.9, 58.75 ± 5.1, 62.5 ± 4.9 ms). Interestingly, Fig. 3c shows a different pattern of neural activity in the barrel cortex of Tg mice. In Tg mice the population response amplitude did not decrease with distance from the center ring, but rather it even showed an opposite behavior and the amplitude becomes even slightly higher as the distance from the barrel’s center gets larger (see additional quantification below). Furthermore, the response dynamic properties of the cortical network are distinctively altered from the network dynamics observed in the Ctrl group (average time-to-peak for the successive rings: mean ± sem; 60 ± 3.6, 61.66 ± 4.01, 66.66 ± 4.2, 68.33 ± 3.07 ms). Moreover, in the Ctrl group, the descending phase of the time course in each ring was separated in time from the other rings, indicating wave propagation into the adjacent barrel columns (average time to half-peak response for the successive rings: mean ± sem; 151.65 ± 18.4, 159.97 ± 22.7, 164.89 ± 26.7, 168.55 ± 19.15 ms). In contrast, in Tg mice, the timing of the descending phase was not distinctly different across the rings (average time to half-maximum amplitude for the successive rings: mean ± sem; 148.79 ± 5.6, 148.66 ± 6.3, 147.3 ± 6.52, 147.70 ± 7.42 ms).

Figure 3d illustrates the spatial spread profile of the VSD signal, measured at 20, 50 and 80 ms after whisker deflection. Each point in the curve represents the average response across all pixels in the corresponding ring. Each point was normalized to the peak response of the inner ring measured at 20 ms after the stimulation onset. At early times, 20 ms after stimulation onset, the spatial spread was similar in both groups. However, throughout the time, the VSD signal showed a significant difference in the area of activation. Single brief whisker stimulation activated significantly larger area in the cortex of Tg mice. The whisker-evoked responses in Tg mice were wider and propagate to much greater distances. Moreover, as mentioned previously, in Tg mice we can clearly see that the amplitude of the population response increases even when the distance from the barrel’s center increases (see Supplementary Figure S1).

The population response differences between Ctrl and Tg mice, in the spatial domain suggested modifications in the properties of the neural network of Tg mice. We wanted to further investigate this result by analyzing the network synchronization. Synchronization of neural activity was hypothesized to play an essential role in encoding the distributed features of a sensory stimulus and propagating information across neuronal circuits^22^. Therefore, we calculated spatial correlation maps during spontaneous activity (see Methods), in the barrel cortex of Crtl and Tg mice. To obtain the spatial correlation maps, we computed zero time-lag Pearson correlations between each pixel in the ROI (i.e. seed pixels) and all surrounding pixels in the near regions, ± 1.25 mm on the x- and y-axes. We then averaged across all correlation maps (each map is aligned on one seed pixel). The outcome of this method represents the mean cross-correlation of *all* pixels in C2 ROI with their nearby surroundings. To investigate whether the cortical synchronization between neuronal populations are driven directly by the stimulation or whether part of the response reflects internal cortical processing^39^, we performed the correlation analyses after subtracting the mean VSD response from each trial and pixel (see Methods).

**Figure 4:**
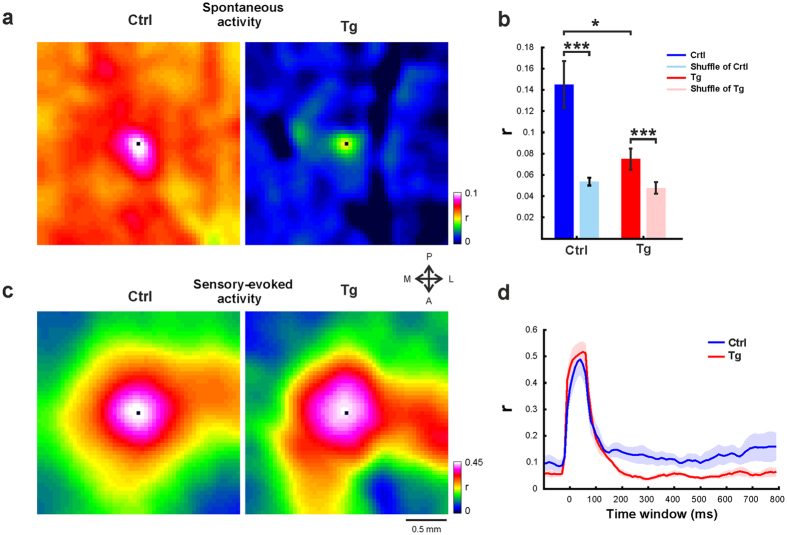
Tg mice exhibited a significant reduction of neural synchrony during the resting state. (**a**) Example of spatial correlation maps in the spontaneous state from two mice. The time averaged spatial correlation maps were computed for pixels located in barrel C2 during spontaneous activity (see Methods for details). Maps were averaged over 2500 ms of spontaneous activity. Left, Crtl; Right, Tg. The color bar depicts the correlation range. (**b**) Spontaneous correlation, grand average analysis. Mean correlation values, for the observed and for the shuffled data, measured at the resting state from the barrel cortex of Crtl (blue colors) and Tg (red colors) mice. The correlations were averaged across mice (Tg n = 6 mice, Crtl n = 8 mice) and over all pixels located up to 200 μm from the central pixel. The correlation values were averaged over 2500 ms of spontaneous activity. In addition, for the spatially shuffled data, the presented correlation values were averaged over all shuffles (n = 100 iterations). Error bar is SEM over mice. *p < 0.05, Wilcoxon rank sum; ***p < 0.001, Wilcoxon signed-rank test. (**c**) Example of spatial correlation maps in the sensory evoked state from two mice. Averaged spatial correlation maps for sensory-evoked activity of pixels located in barrel C2. Left, Crtl; Right, Tg. (**d**) Grand average analysis of correlation across time. The correlation time course were averaged across mice (Tg n = 6 mice, Crtl n = 8 mice) and over all pixels that are located up to 200 μm from the central pixel. Shaded areas represent ± 1SEM across mice.

Figure 4a shows examples of correlation maps from Ctrl and Tg mice. As expected, in Crtl mice, the regions surrounding the barrel C2 ROI, showed higher correlations compared to more distant areas (Fig. 4a, left). The spatial correlation maps indicated that, during the resting state, there is an increased synchronization of neural circuits that are spatially located around the central pixel (i.e. seed pixel), within the barrel field. In contrast, lower synchronization of neural activity has been found in the barrel cortex of Tg mice (Fig. 4a, right). In order to quantify the differences between correlations values, we averaged the correlation at the near surrounding area of the seed pixel over the time. The time interval that was used to calculate the correlation values was 2.5 s for the spontaneous activity. We found that Tg mice exhibited a significant reduction of neural synchrony during the resting state compared with the Ctrl mice (Fig. 4b; mean ± sem; 0.1462 ± 0.02 and 0.0747 ± 0.009 for Ctrl (n = 8) and Tg (n = 6) mice respectively; *p < 0.05, Wilcoxon rank sum). In order to test if the correlation values are an artifact of correlating highly time-varying signals, we shuffled (n = 100 times) the pixels positions in each frame for each trial, before calculating the spatial correlation. Shuffling the pixels within each frame of imaged cortex did not produce these maps, and the correlation values measured in Tg and Ctrl groups were significantly higher than the correlation values measured in the spatially shuffled data (Fig. 4b; ***p < 0.001, Wilcoxon signed-rank). Finally, we computed correlation using a similar approach described in Mohajerani *et al*.^98^ (see Methods). Here, the correlation maps were computed for seed-pixels as before (pixels in C2 ROI) but the spatial correlation window was extended to include the entire imaged area and correlation maps were averaged without aligning on the seed pixel. As expected this analysis yielded similar results, which further reinforced our findings (supplementary Figure S2b; mean ± sem; 0.1158 ± 0.016 and 0.077 ± 0.006 for Ctrl (n = 8) and Tg (n = 6) mice respectively; *p < 0.05, Wilcoxon rank sum).

Next we wanted to investigate the synchronization level during sensory evoked responses (Fig. 4c). The time range used to quantify the correlations values was 150 ms of the sensory-evoked responses (correlation was computed after subtracting the mean evoked VSD response from each trial and pixel; see Methods). Interestingly, we found that the correlation values in Tg mice did not differ significantly, from the correlation values that were measured in Crtl mice (Fig. 4d). Thus, the cortical network in Tg mice showed lower synchronization levels at resting state which lead to abnormal network activities.

Finally, to verify that the neuronal response changes in Tg mice do not emerge from sensitivity difference to anesthetics drugs compared to the Ctrl group, we conducted a series of VSDI experiments using isoflurane anesthesia (see Methods; n = 2 for Tg mice and n = 2 for Ctrl mice). The results obtained under Ketamine/Xylazine or Isoflurane anesthesia were very similar. Under Isoflurane anesthesia the response amplitude to whisker deflection was much higher in the Tg mice compared to the controls (see example maps in Figure S3a,b,d,e, peak response (mean ± sem): 2.8 ± 0.15 × 10^−3^ and 6.4 ± 0.2 × 10^−3^ for Ctrl (n = 55 trials, 2 mice) and Tg mice (n = 64 trials, 2 mice) respectively; ***p < 0.001, Wilcoxon rank sum). Moreover, the spatial spread of the sensory-evoked response was significantly larger in the Tg group (supplementary Figure S3c). Finally, we show that the Tg mice exhibited a significant reduction of neural synchrony during the resting state compared with the Ctrl mice (Figure S3f; synchrony (mean ± sem): 0.076 ± 1.3 × 10^−3^ and 0.065 ± 2.2 × 10^−3^ for Ctrl (n = 40 trials, 2 mice) and Tg mice (n = 60 trials, 2 mice) respectively. ***p < 0.001, Wilcoxon rank sum). Our results are in accordance with previous observations reported in studies using diverse strains of AD mice and various types of anesthesia^18^,^19^,^20^,^29^,^40^.

## Discussion

While many studies have been focused on the effect of the amyloid-β accumulation and plaques at the single cell level, the effects of Aβ on neuronal circuits or complex spatiotemporal dynamics of neuronal networks at the mesoscopic scale with a millisecond time resolution was not well investigated. Here we used *in vivo* VSDI to examine the population response evoked by sensory stimulation in the barrel cortex of Tg and Ctrl mice. We found that in Tg mice, whisker deflection evoked abnormal sensory responses in the barrel cortex. Furthermore, spontaneous activity was more synchronized in the Ctrl animals than in the Tg mice, while synchrony during sensory stimulation reached a similar level.

Our results showed that the whisker-evoked responses in Tg mice had a much higher response amplitude and larger spatial extent, which might be explained by a net increase in network excitability. There is substantial evidence that accumulation of Aβ plaques generates abnormal network excitability^13^,^41^,^42^. Our results are in accordance with previous studies on hyperactivity reported for sensory evoked responses or during spontaneous activity in the olfactory cortices^40^, visual cortex^20^,^43^, hippocampus^17^,^44^,^45^ and frontal cortex of Tg mice^18^,^19^. Augmentation of sensory-evoked hemodynamic responses has been found in APP mice and the authors speculated that the underlying mechanism of the augmented hemodynamics in AD is derived from either increased neuronal activity, increased vascular reactivity, or both^46^. Moreover, the abnormal network excitability was previously associated with reduction in cognitive and sensory processing abilities in Tg mice^20^,^40^. In humans, hyperactivity was reported also in AD patients who showed Aβ plaques accumulation. For example, an fMRI study focused on the default network that was reported to have a reduced activity during memory encoding in normal subjects. Humans with Aβ plaques accumulation showed an aberrant increment in neuronal activity of the default network system during memory encoding^13^,^47^,^48^. In addition, MEG and EEG studies reported that auditory stimulation induced a significant increase of sensory evoked responses in AD patients, compared to normal subjects^49^,^50^. EEG recordings in patients with AD also revealed that visually evoked responses are higher as compared to healthy controls^51^. Finally, hyperexcitability was linked also to epileptiform seizures^13^,^42^. The incidence of epileptiform seizures is increased in AD patients^52^ and epileptic seizure activity has been reported in the cortex and thalamus of mice over expressing APP^15^,^16^.

Our results show that Aβ accumulation and plaques in Tg mice are associated with a significant increase in the spatial extent of the cortical responses inducing abnormal broadening of whisker-evoked responses. The sensory-evoked activity of Tg animals propagates across larger distance in the barrel cortex itself and also outside the barrel cortex to adjacent cortical areas, as compared to Ctrl mice (Figs 2 and 3). The broadening of the evoked response in the barrel cortex of the Tg mice is in accordance with a recent report on broadening of orientation tuning curves in the visual cortex of Tg mice^20^. Our results are also in accordance with a recent study by Hazra *et al*.^45^ who showed that electrical stimulation in hippocampal brain slice evoked a response that spread to a larger extent in Tg animals compared to Ctrl animals. Finally, studies in patients with early stages of AD or individuals at high risk for AD showed that during cognitive tasks, neuronal responses extended over more brain regions relative to normal subjects. It has been speculated that the recruitment of additional brain areas may represent a compensatory mechanism^53^,^54^,^55^,^56^,^57^,^58^. We further propose that the aberrant spread of the cortical activity may not be restricted to sensory stimulation of the barrel cortex, but rather, it may be an emerging property of other sensory cortical areas.

The increase in spatial extent of the evoked cortical responses may be explained by the dynamics of the neuronal responses. Intracellular recordings have revealed that increased variability of responses to stimulation cause a temporal “smear” or broadening of the average response to stimuli^10^. A similar trend can be seen in our results (Fig. 2). The temporal broadening may lead to a spatial increase in activity simply by raising the membrane potentials of the neurons to values closer to threshold.

An important function of lateral inhibition in the barrel cortex is to sharpen the sensory tuning properties, thus each barrel responds maximally to one principal whisker^59^. Beker *et al*.^72^ showed that the Aβ plaques aggregates in the barrel cortex in a non-random pattern, the plaques are concentrated more in the septal areas than inside the barrels. Based on these findings, Beker and colleagues suggested that population responses in the barrel cortex of Tg mice will be much broader compared to Crtl mice. Our data is in accordance with this prediction and demonstrate that Aβ plaques induced abnormal broadening of whisker-evoked responses. In fact, emerging evidence indicates that impairment in the GABAergic system could be a key feature in the pathogenesis of network dysfunction in AD^13^. In the visual cortex of APP mice the hyperactivity of the neurons was associated with a relative decrease in synaptic inhibition^19^. Moreover, electrical recordings from inhibitory interneurons in the dentate gyrus area of APP mouse tissue revealed that they fail to reliably fire action potentials^45^. Recently, *in vitro* whole-cell recordings in the somatosensory cortex of rats, have demonstrated that Aβ abates the GABAergic synaptic transmission through downregulation of GABA_A_ receptors^60^. With regard to the observed neural activity changes in the AD mice, and based on the previous literature, we may speculate that the inhibitory cortical network is impaired in AD.

Synchronous activity is a fundamental feature in the operation of large cortical networks^61^,^62^,^63^,^64^. It has been shown that Aβ accumulation and plaques disrupt the synchrony of convergent inputs and reduce the ability of neurons to successfully integrate and propagate information^10^,^29^. In AD patients, there are large number of evidence for reduced neural synchrony during the resting state^22^. A magnetic resonance imaging study has found that AD is associated with widespread loss of both intra-network and inter-network functional connectivity in resting state. Interestingly, the functional connectivity was more impaired with increasing AD severity^65^. Our results are in accordance with these studies: we found decreased synchronization in the spontaneous state of Tg mice. Furthermore, EEG recording in AD patients revealed a decrease in mean levels of EEG synchronization in the alpha- beta- and gamma-bands^66^,^67^. The reduction in alpha- and beta- bands synchronization correlates with cognitive impairment and the severity of the disease^66^,^68^,^69^. It has been suggested that the reduction in synchrony could be a direct cause of the progressive degeneration of cognitive abilities during the course of the disease^22^.

In the animal model of AD, the results of synchronization are debated. A recent study using the same mouse model used here, reported on decreased synchronization during the spontaneous state^29^. Moreover, using functional connectivity optical intrinsic signal imaging, it has been demonstrated that Aβ deposition is associated with reduced bilateral functional connectivity in multiple brain regions of APP/PS1 mice^14^,^70^. Recently, it has been reported, using large-scale calcium fluorescence imaging in AD mice, that Aβ plaques induce breakdown of long-range coherence of cortical waves^71^. However, another recent study has reported increased levels of alpha- and beta- bands coherence during the spontaneous state^40^. During sensory evoked response we found that synchronization amplitude in Tg mice was larger, and showed similar synchronization level to those of Ctrl mice. In accordance, odor-evoked activity produced an increased synchrony in the piriform cortex of APP Tg mice^40^.

What are the anatomical substrates that may explain the changes in synchronization? In AD patients Aβ deposition increases the curvature of the cortical neurites and lead to abnormal structures^24^. Recently, this was also shown in an AD mouse model^29^. This alteration in the geometry of the neuronal processes could cause changes in the signal transduction properties of the neurons. The increase in the curvature of the neuronal processes correlated with changes in the signal transduction properties of the neurons that lead to synaptic dysfunction^10^. Jitter in timing of convergent inputs can induce prolonged integration time at the postsynaptic neurons. Moreover, the temporal properties of the neurons are a key feature for synchronization of neural networks. The decreased network synchronization and in particular, the suggested dysfunction of the inhibitory network, may further induce wide spread changes such as increased sensory response.

An interesting question is whether the response changes described in this paper emerges from disruption of the anatomical organization and/or morphological alteration in the barrel cortex of APP mice? Cytochrome oxidase staining demonstrated the existence and preservation of the well-known barrel field organization in layer 4 in both Ctrl and Tg mice^72^. This reconciles with the fact that the functional organization of the barrel cortex appears at early stage of brain development (the critical period ends within 16 days after birth)^73^, while the Aβ plaques start to aggregate at much later time (4 months of age)^29^,^72^,^74^. In addition, the early VSD maps (computed at 20–30 ms after whisker stimulation) which were shown to correspond well with the barrel field position and size^32^,^75^ were highly similar for the Ctrl and the Tg mice. These VSD maps reflect the neural processing of early thalamic input to the cortex and the similarity between Ctrl and Tg mice further suggest that this stage of processing was not much affected in Tg mice.

However, Aβ deposition has been associated with various morphological alterations in the cortical tissue, which can lead to morphological disruption of neuronal processing^3^,^4^,^5^,^6^,^7^,^8^,^9^,^21^,^24^,^29^ . Beker *et al*.^72^ provided evidence that Aβ plaques tended to cluster in the septal regions more than in the barrels columns of the barrel cortex. This may suggest that a main impact of Aβ aggregation is on the elaborated inhibitory network of the septal regions. Indeed, we found that although the early sensory responses were similar among Ctrl and Tg mice, the later sensory responses, mediated mainly by neurons in layer 2/3 of the Tg mice, became significantly different compared to the controls. A possible explanation for this is that although neurons in layer 4 may propagate information efficiently into layer 2/3 neurons, the morphological changes in layer 2/3 caused by Aβ plaques aggregation induce population response deficits. Indeed, Beker *et al*.^72^ showed that Aβ plaques are randomly distributed in layer 2/3 which can lead to morphological alteration in neurons and their processes^3^,^4^,^5^,^6^,^7^,^8^,^9^,^21^,^24^,^29^. Moreover a previous study in CA1 pyramidal neurons of APP/PS1 mice showed that structural degeneration and neuronal hyperexcitability were critically linked^21^. In conclusion, we speculate that the at least some of the described functional deficits reflect morphological alterations in layer 2/3 neurons and in the inhibitory interneuron in layer 4 of the barrel cortex. Clearly, more research is needed to fully understand the relation between the changes in population responses and the morphological alternations in the barrel cortex.

To verify that the neuronal response changes in Tg mice do not emerge from sensitivity difference to anesthetics drugs compared to the Ctrl group, we conducted a series of VSDI experiments using isoflurane anesthesia. The results obtained under Ketamine/Xylazine or Isoflurane anesthesia were very similar. Moreover, our findings are consistent with previous studies using diverse strains of AD mice and various types of anesthesia^18^,^19^,^20^,^29^,^40^. Therefore, we can reasonably assume that the influence of anesthesia on our results is minor. Furthermore, we cannot exclude the possibility that the VSD staining quality was different between the two groups which could lead to biased results. However, the early evoked responses in the barrel cortex were rather similar in amplitude and spatial extent, in both groups. Thus, it is logical to consider that the observed differences between the mice groups are not derived from differences in staining quality.

In conclusion, accumulation of Aβ and plaques may disrupt the spatial activity pattern of large-scale networks in the cortex and may lead to functional failures in processing sensory information.

## Materials and Methods

### Animals

Transgenic mice from the B6C3 (APPswe/PS1deltaE9) strain, were used in this study (n = 6)^76^. Their age at the time of recordings was 10–12 months. These transgenic mice express human presenilin 1 (PS1; PS1-dE9 variant) and a chimeric APP (APPswe). Transgenic expression of APP/PS1 and consequent overproduction of Aβ_42_ causes early-onset amyloid deposition in the mice, in which deposits appears as early as 4 months of age. Amyloid burden significantly worsens with age, and by 9 months, the hippocampus and cortex of the transgenic mice are extensively filled with amyloid plaques (see Fig. 1d)^72^,^74^. APP/PS1 transgenic mice express high levels of Aβ and progressively develop many of the pathological phenotypes of AD, including abundant extracellular Aβ plaques, synaptic dysfunction and loss, neuritic abnormalities, astrogliosis and microgliosis, and deficits in the cholinergic transmission^77^,^78^,^79^,^80^,^81^,^82^,^83^,^84^. Moreover, various deficits in cognitive and behavioral tests have been shown in these animals, including impairment in spatial learning and memory, and deficits in long term contextual memory^81^,^84^,^85^,^86^,^87^,^88^,^89^,^90^. However, these mice strain fail to replicate all the aspects of AD, for example they develop memory loss and plaques, with no neurofibrillary tangles and no neuron loss. In conclusion, although APP/PS1 transgenic mice replicate most but not the all human pathology, they are considered to be a useful model of AD disease for studies investigating cognitive, neurophysiological and anatomical changes that are associated with AD disease^82^,^86^,^89^,^91^. Age-matched non-transgenic mice from the same strain (APPswe/PS1deltaE9) were used as control group (n = 8).

### Surgery and staining

All experimental and surgical procedures were carried out according to the NIH guidelines, approved by the Animal Care and Use Guidelines Committee of Bar-Ilan University and supervised by the Israeli authorities for animal experiments. Mice were deeply anaesthetized with an intraperitoneal injection of ketamine (0.13 mg per g body weight) and xylazine (0.01 mg per g). To maintain adequate levels of anesthesia throughout the experiment, supplemental doses of anesthesia were administered intramuscularly as needed. A 5 mm diameter craniotomy was drilled above the entire barrel cortex area of the mouse, and the dura mater was carefully removed. A staining chamber was constructed around the craniotomy with dental acrylic cement, and the brain was stained with voltage sensitive dye (RH-1691; 0.5 mg/ml of artificial cerebrospinal fluid, ACSF) for ~2 hours. After dye staining, the brain was washed with (ACSF) solution, covered with agarose and sealed with a custom cut coverslip. To exclude the possibility that the anesthetic agents may influence the observed results, we conducted a set of VSDI experiments using isoflurane (1.2%) anesthesia (Tg; n = 2 mice, Crtl; n = 2 mice) Isoflurane anesthesia was performed via a mask using a commercially available rodent inhalant anesthesia system (SomnoSuite Small Animal Anesthesia System, Kent Scientific Corporation). Extreme care was taken to verify that the face mask does not touch the whisker during the stimulation.

### Whisker stimulation

Sensory-evoked responses were generated by single brief deflection of whiskers C2 and D1 separately. A single whisker was deflected by a piezoelectric wafer 1–2 mm from the whisker base, at amplitude of 1 mm. The whisker was glued to a thin glass pipette attached to a PIMCA-Bender ceramic insulated piezoelectric actuator. Whiskers were deflected forwards along the anterior-posterior axis of the head.

### Optical imaging using voltage-sensitive dyes

For voltage-sensitive dye imaging we used the MicamUltima system. Images of 100 × 100 pixels (the whole image covers an area of 5^2^ mm^2^; each pixel cover cortical area of 50^2^ μm^2^) were acquired at 100 Hz. During imaging, the exposed cortex was illuminated using an epi-illumination stage with an appropriate excitation filter (peak transmission 630 nm, width at half height 10 nm) and a dichroic mirror (DRLP 650), both from Omega Optical, Brattleboro, VT, USA. In order to collect the fluorescence and reject stray excitation light, barrier post-filter was placed above the diachronic mirror (RG 665, Schott, Mainz, Germany)^28^. To obtain the vascular pattern of the cortex, we imaged the cortex with a green light (540 nm bp10).

### Basic VSDI analysis

All data analysis was performed by using custom-written MATLAB software (version R2011b). The basic analysis of the VSDI signal is detailed elsewhere^27^,^28^,^92^. Briefly, in order to remove the background fluorescence the recorded values at each pixel were normalized by its pre-stimulation activity (dividing by the average VSD signal 30 ms before stimulus onset) at that pixel. The heart beat artifact and the photo bleaching effect were removed by subtraction of the average of blank signal recorded in absence of stimulation. The imaged signals (ΔF/F) reflect relative changes in fluorescence compared to the resting level observed at blank trials.

### Computation of VSD maps

For each recording session the VSD maps were averaged over all trials and the averaged signal was used for further analysis. Pixels located on blood vessels were detected in the blank condition, using a 2D high pass, Gaussian filter in the space domain (σ = 2 pixels). These were excluded from further analysis and they are marked by gray color in the VSD maps. The VSDI maps of the evoked responses were low-pass filtered with a 2D Gaussian filter (σ = 1.5 pixels) for visualization purposes only.

### Defining regions of interests (ROIs)

In order to study the spatial and temporal properties of the VSDI signal in a given area, regions of interests (ROIs) were defined. Because the sensory-evoked responses rapidly spread across the cortex, the early activated area (20 ms after stimulus onset) was used to evaluate the barrel field size. Pixels exceeding a high threshold (75–80% of peak activity in the activated area) were included in the ROI. This threshold reconciled with the expected size of a single barrel (typically containing 15–20 pixels, ~0.05 mm^2^), as was shown in previous studies using a similar approach^75^. All sensory evoked VSD data analyzed in this work and presented in figures were obtained using C2 whisker stimulation. D1 responses were used only in Fig. 1 to visualize the spatial co-localization of the anatomical barrel fields and VSD early evoked responses.

### Spatial profile

To analyze the spread of response profiles derived by whisker stimulation, we first determined the location of the barrel field center by looking for the max response amplitude within the epicenter of the early response. We then divided the area outside the center into 4 non-overlapping ring-like shape ROIs with increasing diameters around the barrel center. The rings size varied from the center up to 800 μm radius at steps of 200 μm (4 pixels) and width of 200 μm (4 pixels) for each ring. We computed the amplitude and time course for each ROI separately by averaging the signal over all pixels in the corresponding ring field (Fig. 3b). The average responses of these ring-like ROIs allowed a direct evaluation of the spatial extent of the evoked response.

### Correlation analysis

#### Spatial correlation maps

We calculated spatial correlation maps for spontaneous and sensory-evoked activity using the following steps. First, each pixel falling within a selected ROI (e.g., C2 barrel field) was centered in a squared spatial matrix (51 × 51 pixels, 2.55 × 2.55 mm). This way we could study spatial correlation patterns extending up to ± 1.25 mm on the x- and y-axes for the pixel located at the center (i.e. the ‘seed pixel’). For each seed pixel in the selected ROI we calculated Pearson correlation coefficient (r) with the remaining pixels in the matrix. Correlations were calculated using an 80 ms sliding window for each trial and pixel in the imaged area. We then aligned all matrices on the seed pixel and averaged across all the correlation maps that were computed separately for each seed pixel. The outcome of this method represents the mean cross-correlation of *all* pixels in C2 ROI with their nearby surroundings (see Fig. 4a). To focus on correlations that reflect internal cortical processing^39^, rather than correlation that are driven directly by the sensory stimulus (i.e. whisker stimulation) the spatial correlation maps were calculated after subtraction of the average VSD response from each VSD single trial, as described in previous studies^92^,^93^,^94^,^95^,^96^,^97^. The averaged (across trials) sensory evoked VSD response was subtracted from each single sensory-evoked trial. A similar procedure was applied for spontaneous trials, and the averaged spontaneous (across trials) VSD response was subtracted from each spontaneous trial. The time range that was used to compute the correlations values was 2500 ms for the spontaneous activity and 150 ms for the sensory-evoked responses. Note that the central pixel, marked by black square in each map, has a correlation value of 1 by definition (this is the correlation of the pixel with itself) and therefore is excluded from further quantification. Finally, to compute the expected correlation value and to test if the correlation values are an artifact of correlating highly time-varying signals, we shuffled the pixels positions in each frame for each trial, and computed the spatial correlation. Shuffling of pixel values within each frame of imaged cortex, before calculating the correlation, did not produce these maps. Moreover, the correlation values that were measured in Tg and Ctrl groups were significantly higher than the correlation values that measured in the spatially shuffled data (Fig. 4b). In addition, we applied a different correlation computation similar to the approach described in a previous study^98^. Here, we did not aligned the maps on the seed pixel, but rather averaged the seed-pixel maps to create an average spatial correlation map (see supplementary Figure S2). In addition we extended the spatial correlation window and computed correlations over the entire imaged area. The maps were low pass filtered using a 2D Gaussian filter (σ = 2 pixels) for visualization purposes only.

#### Time course of correlation

In order to calculate the time course of correlations we averaged the correlation values over all pixels located up to 200 μm from the central pixel in all maps. Each time point represents 80 ms sliding window (±40 ms from the corresponding time point). The correlations signal were than averaged across mice (Tg, n = 6 mice; Crtl, n = 8 mice).

### Statistical analysis

The Tg mice group was compared with the Crtl group using the nonparametric Wilcoxon Rank-Sum test. To determine if the observed correlation values are significantly different from the correlation values that were calculated in the spatially shuffled data we used the Wilcoxon signed-rank test. Alpha levels for statistical significance were set as 0.05. Data are presented as means ± SEM.

### Histology

After imaging, the animals were perfused intracardially with isotonic buffered saline followed by 200 ml 4% paraformaldehyde in phosphate-buffered saline solution. After removal of subcortical structures and cerebellum, the imaged hemisphere was flattened between glass slides to a thickness of ~2 mm. Next, the brain tissue was placed in 30% sucrose and cut into 100 μm slices using a Vibratome. We used cytochrome oxidase staining to identify the barrel cortex structures in layer IV of the somatosensory cortex. The slices were dipped in a solution containing 300 ml of 0.1 M PB, 50 mg of DAB (Sigma-Aldrich), 3 mg of cytochrome *c* (Sigma-Aldrich) and 11 g of sucrose (Bio Lab Ltd.), and incubated overnight at room temperature. Next, The stained brain slices were washed three times by 0.1 M PB for 20 min at room temperature. To visualize the amyloid plaques, the brain sections were stained with Thioflavin-S. Before staining, the brain slices were incubated for 1 h in 50% Ethanol at room temperature. Each slice was covered by a drop of 0.05% Thioflavin-S (Sigma-Aldrich) in 50% Ethanol. Overall, the brain slices were stained for 8 min in the dark. After the Thioflavin-S staining, the sections were washed with two changes of 80% Ethanol and two changes of distilled water^72^. To map the CO image of the barrel cortex to the VSD map (Fig. 1), the very early sensory evoked responses of at least two different whiskers were used as registration points to the corresponding barrel fields in the CO staining image. A similar approach was successfully used by previous studies^99^,^100^,^101^,^102^.

## Additional Information

**How to cite this article**: Ma’atuf, Y. *et al*. Abnormal Population Responses in the Somatosensory Cortex of Alzheimer's Disease Model Mice. *Sci. Rep.* **6**, 24560; doi: 10.1038/srep24560 (2016).

## Supplementary Material

Supplementary Information
