# Supplementary material for: Abnormal Population Responses in the Somatosensory Cortex of Alzheimer’s Disease Model Mice
Source: Sci Rep. 2016 Apr 15;6:24560. doi: 10.1038/srep24560 (PMC4832196; doi:10.1038/srep24560)
Supplement: Supplementary Information [file srep24560-s1.pdf]

Supplementary Information

Title:

Abnormal Population Responses in the Somatosensory Cortex of Alzheimer's Disease Model Mice.

Authors:

Yossi Ma'atuf[*1], Edward A. Stern[2,3], Hamutal Slovin[2]

Affiliation:

1. The Mina and Everard Goodman Faculty of Life Sciences, Bar-Ilan University, Ramat-Gan 52900, Israel

2. The Gonda Multidisciplinary Brain Research Center, Bar-Ilan University, Ramat Gan, 52900 Israel

3. MassGeneral Institute of Neurodegenerative Disease, Department of Neurology, Massachusetts General Hospital, Charlestown, Massachusetts 02129

Supplementary Figures S1-3

Figure S1:

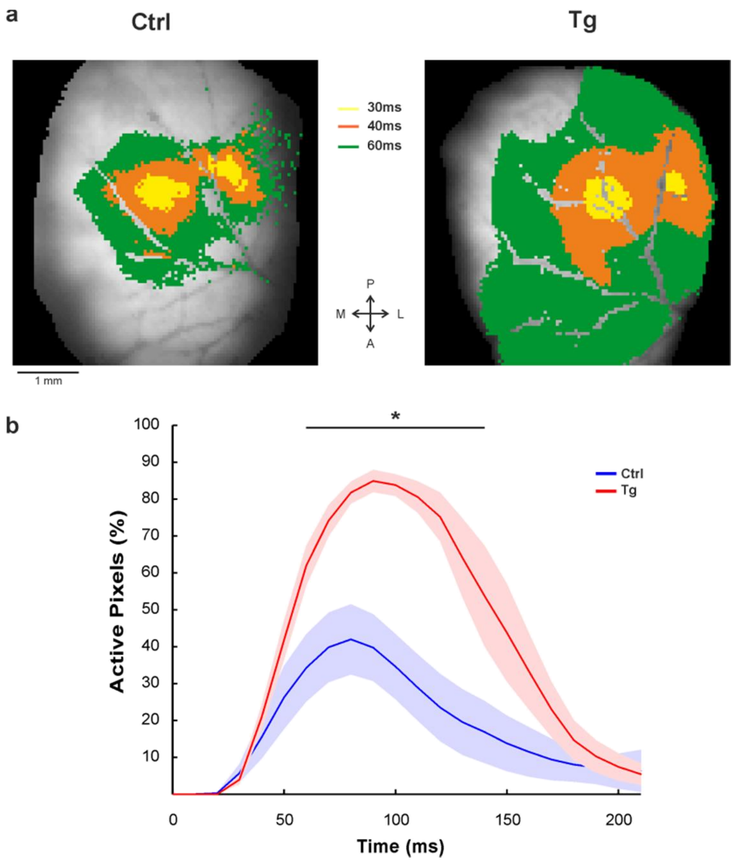

Figure S1. The spread of the VSD response in the barrel cortex of Tg mice is much larger. a: Example maps of high response regions in the barrel cortex of Ctrl (left) and Tg (right) mice. The maps show the spatial distribution of pixels with high evoked response, which were defined as exceeding a threshold of 50% of peak response at 30, 40 and 60 ms after stimulus onset (yellow, orange and green, respectively). Only pixels exceeding this threshold were included in this analysis. Left, Crtl; Right, Tg. **b:** The fraction of pixels within the entire imaged area, exceeding threshold of 50% of peak, as a function of time. The responses were averaged across mice (n=6 Tg mice; n=8 Ctrl mice). Error bars represent ± 1SEM across mice. * p<0.05 , Wilcoxon rank sum.

Figure S2:

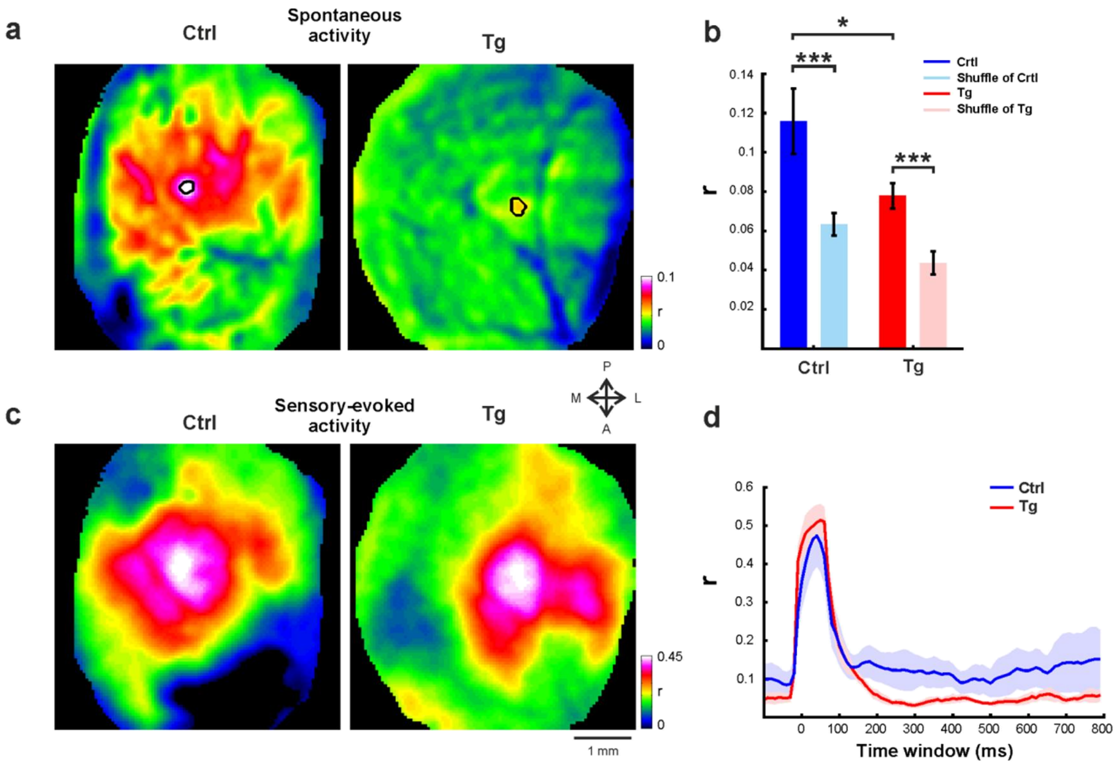

Figure S2. Tg mice exhibited a significant reduction of neural synchrony during the resting state. a: Example of spatial correlation maps in the spontaneous state from two mice. The time averaged spatial correlation maps were computed for pixels located in barrel C2 during spontaneous activity (see Methods for details). Maps were averaged over 2500 ms of spontaneous activity. Left, Crtl; Right, Tg. The color bar depicts the correlation range. The black contour line represents the C2 ROI **b:** Spontaneous correlation, grand average analysis. Mean correlation values, for the observed and for the shuffled data, measured at the resting state from the barrel cortex of Crtl (blue colors) and Tg (red colors) mice. The correlations were averaged across mice (Tg; n=6 mice, Crtl; n=8 mice) and over all pixels located in barrel C2 ROI. The correlation values were averaged over 2500 ms of spontaneous activity. In addition, for the spatially shuffled data, the presented correlation values were averaged over all shuffles (n=100 itterations). Error bar is SEM over mice. * $p<0.05$, Wilcoxon rank sum; *** $p<0.001$, Wilcoxon signed-rank test. **c:** Example of spatial correlation maps in the sensory evoked state from two mice. Averaged spatial correlation maps for sensory-evoked activity of pixels located in barrel C2. Left, Crtl; Right, Tg. **d:** Grand average analysis of correlation across time. The correlation time course were averaged across mice (Tg; n=6 mice, Crtl; n=8 mice) and over all pixels that are located in barrel C2 ROI. Shaded areas represent ± 1SEM across mice.

Figure S3:

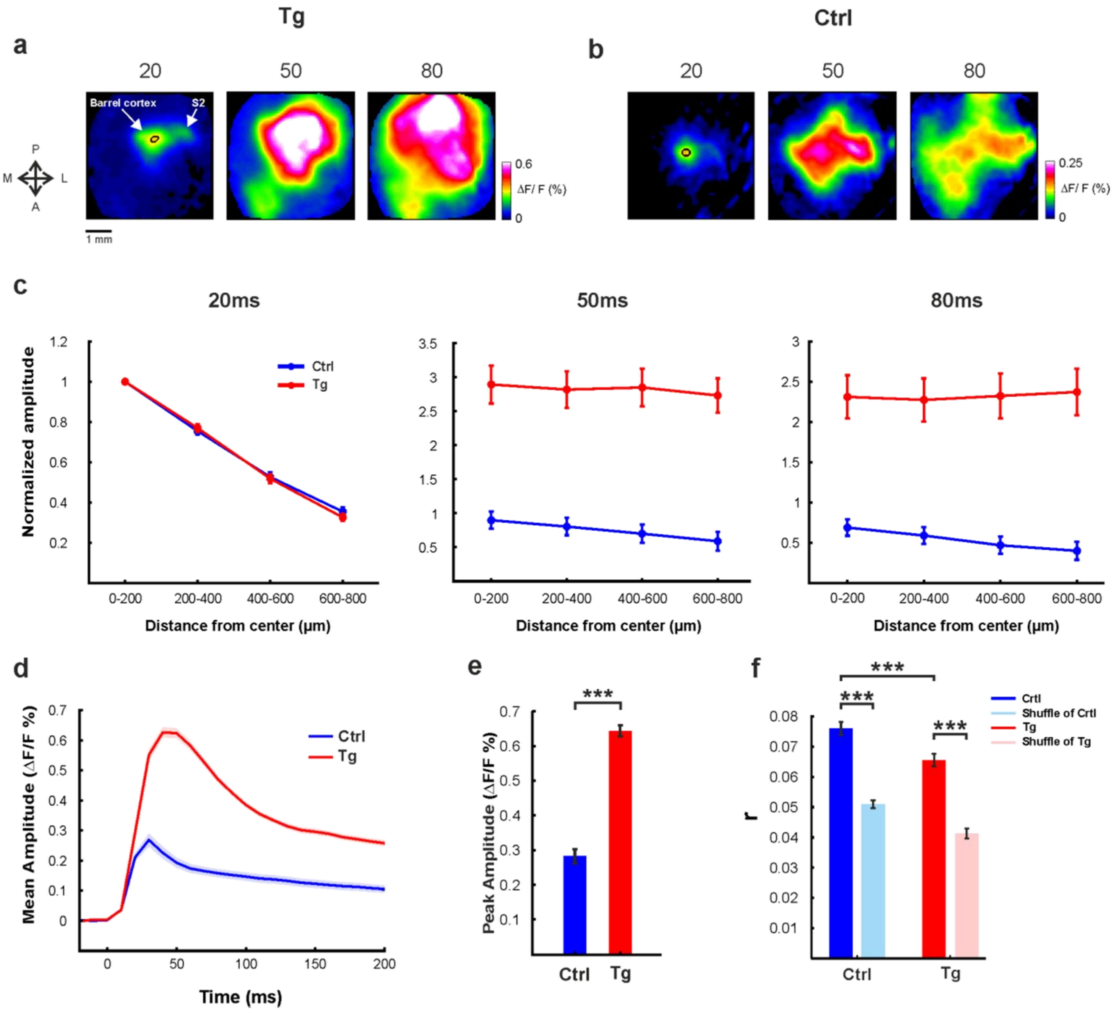

Figure S3. VSD responses in the barrel cortex under isoflurane anesthesia. a-b: Population response maps, evoked by brief C2 whisker deflection, in the barrel cortex of Ctrl (right; example session) and Tg (left; example session) mice. The numbers above the maps represent the time in ms after whisker stimulation onset. Note the colorbar on the right and ΔF/F range that is much larger for the Tg mouse. The VSD maps were low-pass filtered with a 2D Gaussian filter (σ=1.5 pixels) for visualization purposes only. The black contour line on the maps depicts the C2 ROI. **c:** The normalized amplitude, at 20, 50 and 80 ms after stimulation onset, as a function of the distance from the barrel center. Error bars represent ± 1SEM across trials (Tg; n=55, 2 mice, Crtl; n=64, 2 mice). **d:** Time course of the VSD response, averaged across trials. Time course of the VSD response in C2 ROI of Crtl and Tg mice (ROIs are depicted as black contours in a and b maps). The responses were averaged across trials (n=55, 2 Tg mice; n=64, 2 Ctrl mice). Whisker stimulation is at t=0. Shaded areas represent ± 1SEM across trials. **e:** Peak response amplitude evoked by brief whisker

deflection, averaged across all trials (Tg; n=55 mice, Crtl; n=64 mice). *** $p<0.001$, Wilcoxon rank sum. **f:** Correlation maps on the spontaneous state, grand average analysis. Mean correlation values, for the observed and for the shuffled data, measured at the resting state from the barrel cortex of Crtl (blue colors) and Tg (red colors) mice. The correlations were averaged across the spontaneous activity trials (Tg; n=60, 2 mice, Crtl; n=40, 2 mice) and over all pixels located in barrel C2 ROI. The correlation values were averaged over 2500 ms of spontaneous activity. In addition, for the spatially shuffled data, the presented correlation values were averaged over all shuffles (n=100 iterations). Error bar is SEM over trials; ***$p<0.001$ Wilcoxon rank-sum test for comparison between Tg and Ctrl mice; ***$p<0.001$; Wilcoxon signed-rank test for comparison between the correlation values and the spatially shuffled data.
